# Beyond Seizures as an Outcome Measure: A Global Severity Scoring System for CDKL5 Deficiency Disorder

**DOI:** 10.1002/brb3.71061

**Published:** 2025-11-14

**Authors:** Peter Jacoby, Eric D. Marsh, Scott Demarest, Jacinta M. Saldaris, Helen Leonard, Heather E. Olson, Joni N. Saby, Elia Pestana‐Knight, Rajsekar Rajaraman, Dana Price, Judith Weisenberg, Bernhard Suter, Jenny Downs, Tim A. Benke

**Affiliations:** ^1^ Centre for Child Health Research, The Kids Research Institute Australia The University of Western Australia Perth Western Australia Australia; ^2^ Division of Child Neurology, Departments of Neurology and Pediatrics Children's Hospital of Philadelphia, Perelman School of Medicine, University of Pennsylvania Philadelphia Pennsylvania United States; ^3^ Department of Pediatrics and Neurology, School of Medicine, University of Colorado, Precision Medicine Institute Children's Hospital Colorado Aurora Colorado United States; ^4^ Division of Epilepsy and Clinical Neurophysiology and Epilepsy Genetics Program, Department of Neurology Boston Children's Hospital Boston Massachusetts United States; ^5^ Cleveland Clinic Neurological Department Epilepsy Center Cleveland Ohio United States; ^6^ UCLA Mattel Children's Hospital Los Angeles California United States; ^7^ NYU Langone Health and Department of Neurology New York University New York New York United States; ^8^ St. Louis Children's Hospital and Washington University School of Medicine St Louis Missouri United States; ^9^ Department of Pediatrics & Neurology Baylor College of Medicine Houston Texas United States

**Keywords:** biomarkers, CDKL5 deficiency disorder, epilepsy, healthcare, outcome assessment, quality of life

## Abstract

**Background**: CDKL5 deficiency disorder (CDD) is a rare developmental and epileptic encephalopathy (DEE) associated with multiple impairments and comorbidities. Outcome measures for disease‐modifying clinical trials for DEEs should measurably capture a spectrum of caregiver priorities and be externally validated.

**Methods**: The International CDKL5 Clinical Research Network was the data source for this observational study. A Structural Equation Model was constructed with latent, exogenous variables related to observed clinical features to calculate a global severity score from the following assessments: the CDKL5 Clinical Severity Assessment‐Clinician and ‐Caregiver, Communication and Symbolic Behavior Scales Developmental Profile Infant Toddler Checklist and the Sleep Disturbance Scale for Children. Quantitative EEG power was measured as a biomarker. The Quality of Life Inventory—Disability measured quality of life.

**Results**: Acceptable fit statistics for models were obtained using data from 206 subjects (median [range] age 6.8 [3 months to 40] years). Motor and communication measures were the most important weighted contributors to the global severity score which correlated well with the biomarker and quality of life to support external validation.

**Conclusions**: The resultant global severity score provided evidence that the assessments formed a coherent set of measures that reliably and meaningfully captured the diversity of severity in CDD. The models illustrated how the symptoms form a measurable network of relationships which may be suitable as an outcome measure for CDD and DEEs more broadly in clinical trials.

## Graphical abstract

A validated global measure of severity for CDKL5 deficiency disorder was obtained from structural equation modeling that enables clinical trial readiness.

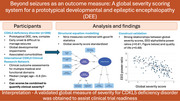

## 1 Introduction

CDKL5 deficiency disorder (CDD) is a rare developmental epileptic encephalopathy (DEE) (Symonds et al. 2019). CDD is characterised primarily by early onset, medication refractory seizures and early, severe, and persistent cognitive, motor and social impairments. Affected individuals also have frequent sleep disturbances, cortical visual impairment (CVI), abnormal muscle tone, and systemic issues that include gastrointestinal, feeding and orthopedic problems, making CDD a prototypical DEE as it broadly encompasses severe features typically seen across most DEEs (Leonard et al. 2022; Scheffer et al. 2025; Scheffer et al. 2024).

Advances in molecular and cellular techniques to manipulate gene expression along with the discovery of causative genes for many individuals with severe early onset epilepsies has led to intensive efforts to generate new genetic medicines (Knowles et al. 2022). For example, the phenotype of the CDKL5 animal model can be improved via protein replacement (Trazzi et al. 2018) and gene replacement (Gao et al. 2020). Clinical trials of precision therapies for CDD are imminent and it is imperative that suitable clinical outcome assessments (COAs) are available to demonstrate their impacts.

The lack of gold‐standard COAs suited to the spectrum of symptoms and severity of CDD is due, in part, to little previous emphasis on quantitative assessment and follow‐up of children and adults with severe or profound intellectual disabilities. Existing clinical measurements evaluate cognitive, motor, or social difficulties well for children who have a mild to moderate impairment but they almost all have a floor effect when attempting to measure more severe impairments. (Saldaris et al. 2024) We established the International CDKL5 Clinical Research Network (ICCRN, NCT05558371), an umbrella working group that includes the International CDKL5 Disorder Database (ICDD) (Leonard et al. 2022) and the CDD Center of Excellence (COE) network comprising US academic institutions with specific clinical expertise and research interest in CDD (Demarest et al. 2019). The purpose of the ICCRN is to generate, evaluate, and validate a suite of COAs initially for CDD but likely applicable to other severe DEEs. Our team has successfully modified, refined, and validated measures of neurologic symptoms associated with CDD (Saldaris et al. 2021; Ziniel et al. 2023; Saldaris et al. 2024), functional abilities (Saldaris et al. 2024; Saldaris et al. 2022; Saldaris et al. 2024), and quality of life (Saldaris et al. 2023) (summarized in Table S1). We are also testing EEG and visual and auditory evoked potentials as biomarkers of clinical severity (Saby et al. 2022). We have modified and validated multiple novel COAs that capture different domains of functioning and perspectives on severity (parent/caregiver‐report and clinician‐report). However, regulatory approvals that favor a single COA are challenged by the need to encompass multiple, interacting and complex domains of feeling and functioning that are prioritized by caregivers. Parents with a child with CDD have identified seizures, developmental impairments, poor vision, and sleep all as priority symptoms in need of new therapeutics (Mingorance et al. 2020; Neul et al. 2023), domains that reflect the complexity and severity of the CDD phenotype. For trial outcomes to be fit‐for‐purpose, COAs need to be reliable, valid, sensitive to change and able to capture multiple priority aspects of DEEs in order to be fully responsive to caregiver priorities.

Using Structural Equation Modeling, we aimed here to investigate how our suite of measures of functional abilities and comorbidities combined to reflect a single latent construct of global severity in CDD. We determined the test–retest reliability of the global severity score and, for external validation, we then individually evaluated the relationships between global severity, EEG parameters, and quality of life.

## 2 Materials and Methods

### 2.1 Data Sources

The ICCRN including the ICDD (Leonard et al. 2022) and the CDD COE network (Demarest et al. 2019) were the data sources for this study (NCT05558371). Parent/caregivers registered with the ICCRN and whose child had a CDKL5 variant considered to be pathogenic or likely pathogenic were invited to participate.

Families who attended a US COE (Children's Hospital Colorado/University of Colorado School of Medicine, Boston Children's Hospital, Children's Hospital of Philadelphia, St. Louis Children's/Washington University School of Medicine, NYU Langone Health, Cleveland Clinic, UCLA, Baylor College of Medicine) between January 2021 and February 2023 were invited to participate in a clinic evaluation and to complete parent‐reported questionnaires to describe their child's epilepsy, other comorbidities, communication, and quality of life. Families who were registered with the ICDD who had not attended a COE clinic, including families living outside the United States, were invited to complete the parent‐report questionnaires between August 2022 and February 2023. As several questionnaires were still in development and had not yet been translated to languages other than English, a primary caregiver needed to be fluent reading and speaking English as a requirement for participation. Clinician‐ and parent‐reported data were collected and managed using REDCap electronic data capture tools (Harris et al. 2009) hosted at the University of Colorado and The Kids Research Institute Australia.

Families attending a US COE were also invited to participate in a longitudinal study commencing January 2023. This comprised clinical evaluations and questionnaire administration every 6 months over a period of 2 years and included collection of data on a randomly selected subgroup of participants on two occasions separated by approximately 4 weeks.

Ethics approval for this study was provided by the Human Research Ethics Committees at the University of Colorado (COMIRB 19–2756) and University of Western Australia (RA/4/20/6198). Primary caregivers provided informed written consent to participate.

### 2.2 Outcome Measures

#### 2.2.1 Clinician Assessment

1. **CDD Clinical Severity Assessment (CCSA‐Clinician)**—The CCSA‐Clinician was modified from the CDKL5 Clinical Assessment (Demarest et al. 2019) using cognitive interviewing and nominal group technique (Saldaris et al. 2021). Factor analysis of data from 148 individuals indicated 3 domains: Communication (4 items), Vision (5 items), and Motor (5 items) with high factor loadings, good internal consistency and model fit, and excellent item inter‐ and intra‐rater clinician reliability via a video‐based learning module (Saldaris et al. 2024). Items relating to movement disorders, including chorea and stereotypies, did not fit into the validated factor structure for CDD and were not included in the suite of measures.

#### 2.2.2 Parent Reported Questionnaires

2. **CDD Clinical Severity Assessment (CCSA‐Caregiver)**—The CCSA‐Caregiver was also modified from the CDKL5 Clinical Assessment (Demarest et al. 2019) using cognitive interviewing (Ziniel et al. 2023). Factor analysis of 198 datasets indicated that 18 items loaded to four domains: Seizures (5 items), Alertness (6 items), Behavior (4 items), and Feeding (3 items). Item factor loadings were high, internal consistency was good, and there was evidence of known groups validity and excellent test–retest reliability (Saldaris et al. 2024).
3. **Communication and Symbolic Behavior Scales Development Profile Infant Toddler Checklist (CSBS‐DP ITC)**—The CSBS‐DP ITC is a 24‐item parent‐reported scale that evaluates early communication skills (Wetherby et al. 2002). The one factor model which yields a single total score had satisfactory convergent and known groups validity (*n* = 150) and excellent test–retest reliability (*n* = 73) (Saldaris et al. 2024).
4. **Sleep Disturbance Scale for Children (SDSC)**—The SDSC is a pediatric sleep questionnaire (Bruni et al. 1996), which comprises 26 items that load to six domains. We administered the “Disorders of Maintaining Sleep” (DIMS) and “Disorders of Excessive Somnolence” (DOES) domains because of their clinical relevance to CDD. For individuals aged 3 years and older (*n* = 125), confirmatory factor analysis indicated removal of two items in the DIMS domain and one item in the DOES domain. Factor loadings, internal consistency, and indices of relative fit were satisfactory, and criteria for divergent validity were met (Saldaris et al. 2024).
5. **Quality of Life Inventory—Disability (QI‐Disability)—**QI‐Disability is a 32‐item scale, which measures parent‐observed child behaviors across six domains. QI‐Disability has been previously validated in intellectual disability and CDD. Confirmatory factor analysis verified the 6‐factor structure for CDD where model fit statistics were mostly satisfactory although the average variance extracted (AVE) values were less than the maximum correlation squared value for the Social Interactions and Independence domains, indicating that divergent validity criteria for these domains were not met (Saldaris et al. 2023). Test–retest reliability scores were good and scores were responsive to changes in physical health status (Saldaris et al. 2023).

#### 2.2.3 Electrophysiological Assessments

Electrophysiological Assessments as Potential Biomarkers of Clinical Severity or Change—Quantitative EEG parameters, in particular alpha/delta and theta/delta power ratios, were calculated on EEG recorded while the subjects were awake and resting as we have previously reported (Saby et al. 2022). These EEG features, which are measures of broad brain function, have been demonstrated to correlate with clinical severity in Rett syndrome, CDD, MECP2 duplication syndrome, and FOXG1 (Saby et al. 2022; Saby et al. 2020; Saby and Marsh 2025; Saby et al. 2024).

### 2.3 Statistical Methods

A reflective model of CDD was investigated, with latent severity variables posited as common causes of observed clinical features. A structural equation model (SEM) was constructed including the following nine observed exogenous variables:

- CCSA‐Clinician—Motor, Vision and Communication domain scores.
- CCSA‐Caregiver—Seizures, Alertness and Feeding domain scores.
- CSBS‐DP ITC total communication scores.
- SDSC—DIMS (Insomnia) and DOES (Daytime Sleepiness) domain scores.

**FIGURE 1:**
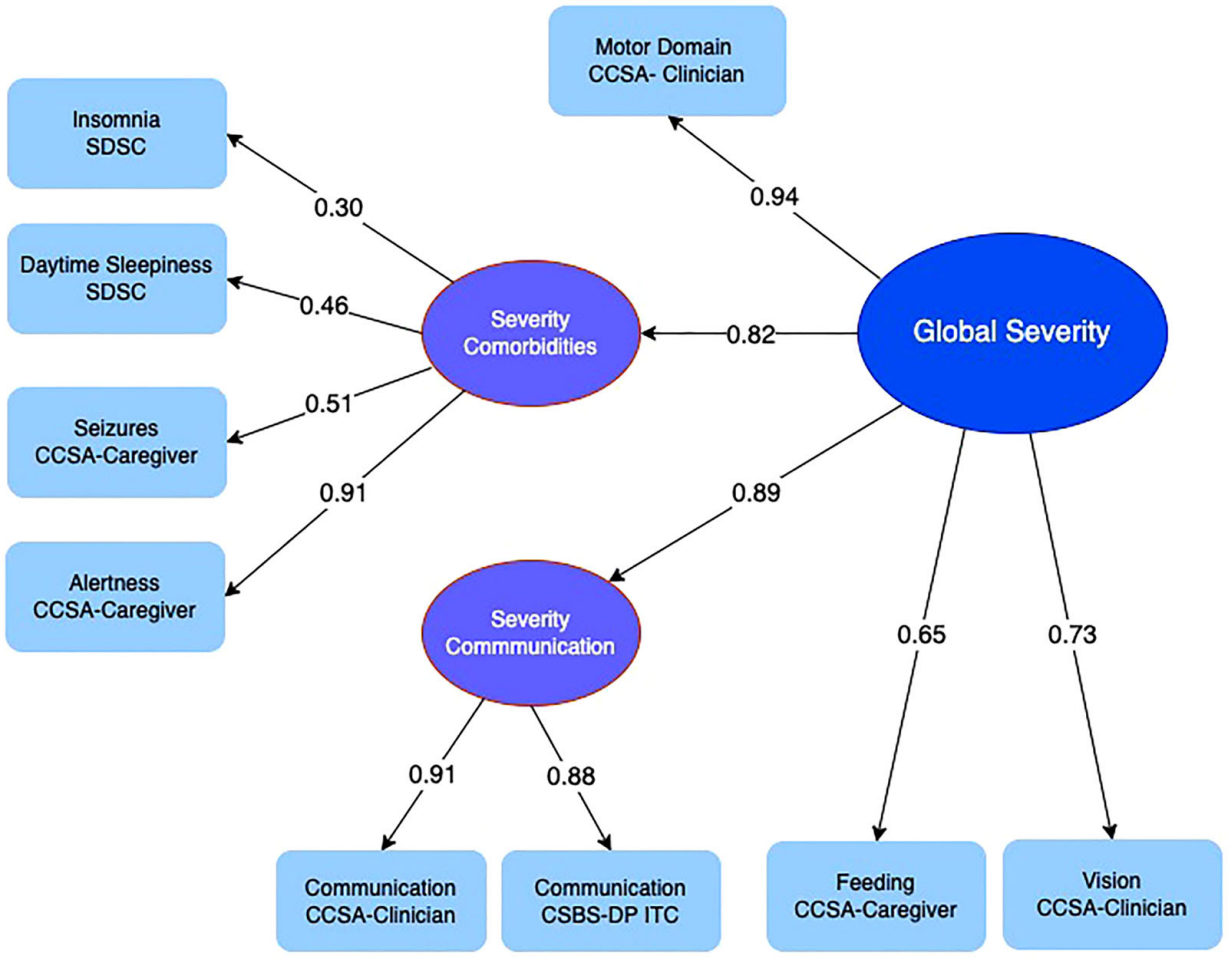
**Structural equation model of severity in CDD showing sources of data and factor loadings**. The model included two latent exogenous variables (Communication and Comorbidities) representing an a priori grouping of different aspects of severity, and one latent endogenous variable representing global severity. Using Full Information Maximum Likelihood (FIML) to use all available data in the presence of missing observations, fit statistics, and factor loadings were documented and a global severity score calculated for each participant for each model. Multiple linear regression analysis was performed, using complete case analysis, with global severity score as the dependent variable and the observed variables included in the SEM model as independent variables. The regression coefficients were scaled to sum to one so that the severity score has the same metric as the individual component scores. These coefficients form a set of weightings that were applied to estimate a global severity score for each CDD subject. Model fit statistics indicated good fit demonstrated by the chi‐square/df (1.21), root mean square error of approximation (RMSEA, 0.031), Comparative Fit Index (CFI, 0.992) and Tucker–Lewis Index (TLI, 0.998) values, which were all within the recommended range (Hu and Bentler 1999).

The model (Figure 1) included two latent exogenous variables (Communication and Comorbidities) representing an a priori grouping of different aspects of severity, and one latent endogenous variable representing global severity.

The model was fitted using Full Information Maximum Likelihood (FIML) to use all available data in the presence of missing observations. Data was used from all participants who had two or more of the assessment scores reported. For each model, fit statistics and factor loadings were documented and a global severity score calculated for each participant. The global severity scores were standardized to a mean of zero and a standard deviation of one. Higher severity scores equated to greater clinical severity.

Subsequently, a multiple linear regression analysis was performed, using complete case analysis, with global severity score as the dependent variable and the observed variables included in the SEM model as independent variables. The regression coefficients were scaled to sum to one in order that the severity score has the same metric as the individual component scores. These coefficients then form a set of weightings, which could be applied to estimate a global severity score for any person with CDD via a weighted average of observed COA scores.

Alpha/Delta and Theta/Delta power ratios from those subjects who had EEG results recorded, behavior domain scores from the CCSA‐Caregiver, and QI‐Disability scores were independently correlated with the global severity score using the Pearson's correlation coefficient as done previously (Saby et al. 2022; Saby et al. 2020; Saby and Marsh 2025; Saby et al. 2024). Behavior was considered separately to the concept of clinical severity because of the phenomenon where greater clinical severity can be associated with more challenging behaviors in genetic epilepsy conditions (Besag et al. 2016).

The weightings were used to calculate global severity scores for the participants in the test–retest reliability subgroup. Reliability was assessed for global severity and its nine COA components, via the Intra‐Class Correlation (ICC) with values interpreted as indicating good (0.75–0.9) or excellent (>0.9) reliability.

All analyses were conducted in STATA (StataCorp. 2021. Release 17).

## 3 Results

There were 206 subjects (149 attended a COE, 57 were uniquely recruited through the ICDD), with at least two completed COAs and whose data was used in fitting the SEM. Missing participant data ranged from 4% for the CCSA‐Caregiver domains to 39% for the sleep questionnaire domains (Table 1). The median age was 6.8 years (range 3 months to 40 years). Most (82%) of the participants were female. The majority used non‐verbal communication methods (76.9%) or were unable to walk (56.1%). Demographic details and functional ability of the subjects are described in Table 1 together with scoring details on the nine observed variables used in the SEM.

**TABLE 1:** Characteristics of participants and scores on observed variables (*N* = 206).

Variable	Category	*N* (%)
Age group *N* = 206	Under 7 years	106 (51.5)
	7 years and over	100 (48.5)
Sex *N* = 206	Male	37 (18.0)
	Female	169 (82.0)
Communication (≥12 m), *N* = 182	Non‐verbal	140 (76.9)
	Some words	42 (23.1)
Mobility (≥18 m), *N* = 171	Unable to walk	96 (56.1)
	Walks with assistance	34 (19.9)
	Walks without assistance	41 (24.0)
**Data source**	**Observed variable**	**Mean (SD)**
Sleep questionnaire^a^ (maximum score 100) *N* = 125 (39% missing)	Insomnia	39.6 (22.1)
	Sleepiness	34.4 (21.6)
Communication questionnaire^b^ (maximum score 57) *N* = 164 (20% missing)	CSBS‐DP ITC	15.4 (13.9)
Clinician CCSA^a^ (maximum score 100) *N* = 148 (28% missing)	Vision domain	40.2 (26.1)
	Communication domain	67.3 (24.4)
	Motor domain	60.1 (28.9)
Caregiver CCSA^a^ (maximum score 100) *N* = 198 (4% missing)	Seizures domain	35.5 (24.7)
	Alertness domain	35.6 (20.9)
	Feeding domain	36.5 (33.5)
Quality of life questionnaire^b^ (maximum score 100) *N* = 145 (30% missing)	QI‐Disability Total Score	60.8 (15.9)

*Note*: Demographic details and functional ability of subjects are described with scoring details on the nine observed COAs used in the SEM.

^a^ Higher scores indicate better functioning.

^b^ Higher scores indicate greater severity.

**FIGURE 2:**
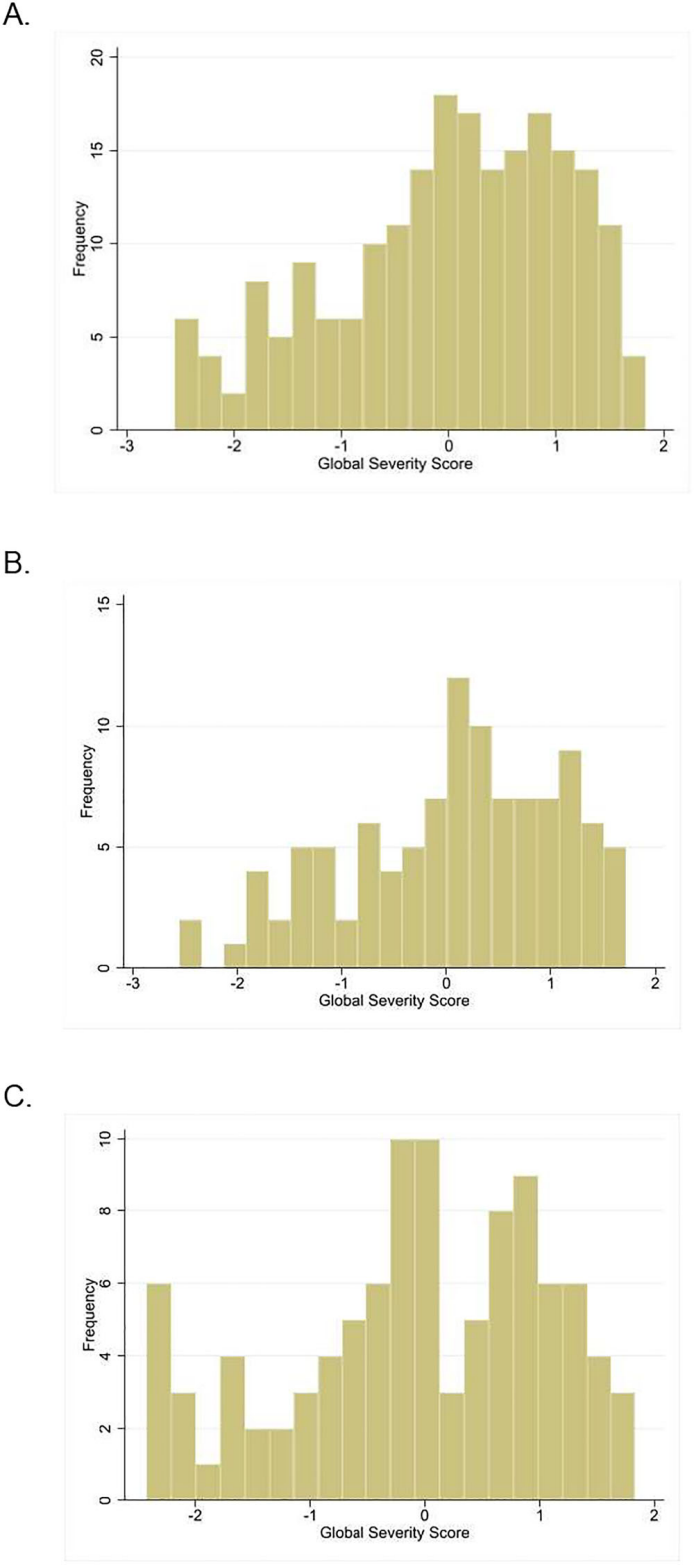
**Distribution of global severity scores**. A, The distribution of global severity scores is shown for all children (A, *N* = 206), for those aged less than 7 years (B, *N* = 106) and those aged 7 years and over (C, *N* = 100). The age‐segregated distributions were not significantly different (*p* (*t*‐test) = 0.39). Each distribution has a slight skew. The global severity scores were standardized to a mean of zero and a standard deviation of one. Higher severity scores equated to greater clinical severity.

Model fit statistics indicated good fit demonstrated by the chi‐square/df (1.21), root mean square error of approximation (RMSEA, 0.031), Comparative Fit Index (CFI, 0.992), and Tucker–Lewis Index (TLI, 0.998) values, which were all within the recommended range (Hu and Bentler 1999). Factor loadings are shown in Figure 1. Many children with CDD demonstrate a plateau of developmental skills around age 7 (Demarest et al. 2019; Fehr et al. 2015). The distribution of global severity scores is shown in Figure 2A for all children (*N* = 206), for those aged less than 7 years (Figures 2B, *N* = 106) and those aged 7 years and over (Figures 2C, *N* = 100). Each distribution has a slight skew. The age‐segregated distributions were not significantly different (*p* (*t*‐test) = 0.39).

**FIGURE 3:**
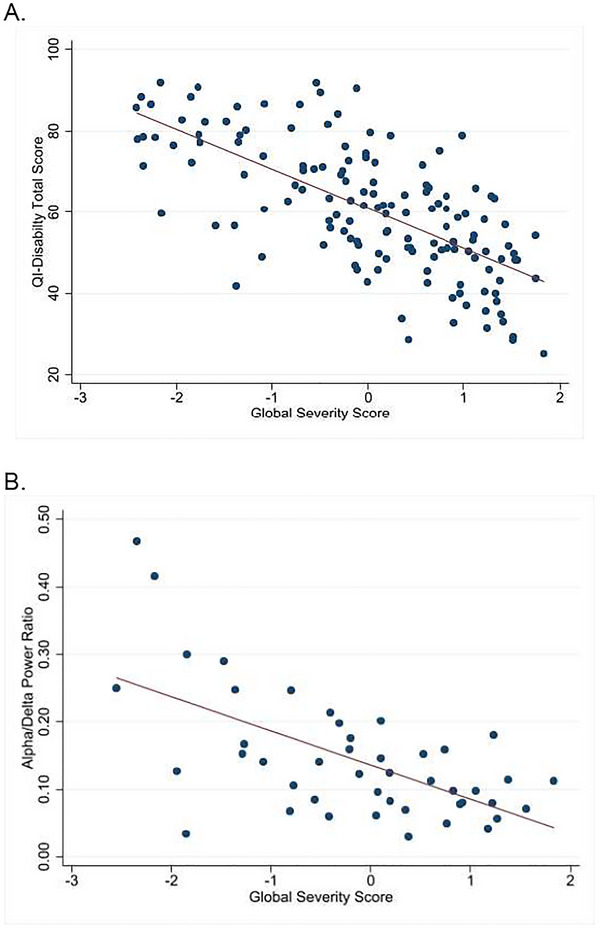
**Relationship of alpha/delta EEG power ratios and quality of life scores to global severity scores**. (A) Global severity scores demonstrated moderately linear high correlation (solid line) with QI‐Disability total score (*N* = 145, Pearson's correlation coefficient = 0.68). The global severity scores were standardized to a mean of zero and a standard deviation of one. Higher severity scores equated to greater clinical severity. (B) Global severity scores demonstrated moderately high linear correlation (solid line) with Alpha/Delta power ratios (*N* = 45, Pearson's correlation coefficient = 0.61).

There was a moderately high correlation between global severity scores and QI‐Disability total score (*N* = 145) with a Pearson's correlation coefficient of 0.68 (Figure 3A). However, there was a small negative correlation (Pearson's coefficient, –0.21) between global severity and the behavior domain score of the CCSA‐Caregiver questionnaire (not shown). Forty‐five individuals had quantitative EEG assessments. There was a moderately high correlation between global severity scores from the model and Alpha/Delta power ratios with Pearson's correlation coefficient of 0.61 (Figure 3B).

Table 2 shows the calculated weightings applied to the component COA scores, assuming all scores are available, to arrive at an estimate of global severity. The weightings are normalized to sum to one, with the assumption that all the scores are normalized to the same scale. The motor domain of CCSA‐Clinician has the highest weighting with the communication scales and the alertness domain of CCSA‐Caregiver also having high weightings.

**TABLE 2:** Weightings applied to clinical outcome scores for calculation of global severity.

Clinical outcome assessment/domain		Weight^a^
Motor	Motor domain—CCSA‐Clinician	0.467
Comorbidities	Insomnia—SDSC	0.007
	Daytime sleepiness—SDSC	0.012
	Seizures domain—CCSA‐Caregiver	0.012
	Alertness domain—CCSA‐Caregiver	0.111
Communication	Communication domain—CCSA‐Clinician	0.105
	Communication—CSBS‐DP	0.142
Vision	Vision domain—CCSA‐Clinician	0.091
Feeding	Feeding domain—CCSA‐Caregiver	0.052

*Note*: Calculated weightings applied to the component COA scores, assuming all scores are available, are normalized to sum to one, with the assumption that all the scores are normalized to the same scale.

^a^ Assumes that all scores are normalized to the same scale.

There were 18 participants in the longitudinal study who had complete test–retest reliability sets of COA data, separated by an interval of approximately 4 weeks, enabling test–retest reliability estimation. The global severity score had excellent reliability (ICC = 0.98) while the nine component COAs all had good or excellent reliability (ICC > 0.75) (Table 3).

**TABLE 3:** Intra‐class correlations (ICC) indicating test–retest reliability of clinical outcome scores and of global severity.

Clinical outcome assessment/domain		ICC (95% CI)
Motor	Motor domain—CCSA‐Clinician	0.93 (0.83–0.97)
Comorbidities	Insomnia—SDSC	0.83 (0.61–0.93)
	Daytime sleepiness—SDSC	0.87 (0.69–0.95)
	Seizures domain—CCSA‐Caregiver	0.89 (0.73–0.96)
	Alertness domain—CCSA‐Caregiver	0.81 (0.56–0.93)
Communication	Communication domain—CCSA‐Clinician	0.92 (0.79–0.97)
	Communication—CSBS‐DP	0.98 (0.95–0.99)
Vision	Vision domain—CCSA‐Clinician	0.88 (0.70–0.95)
Feeding	Feeding domain—CCSA‐Caregiver	0.99 (0.99–1.00)
		
Global severity		0.98 (0.95–0.99)

*Note*: Eighteen participants in the longitudinal study completed test–retest reliability sets of COA data, separated by an interval of approximately 4 weeks, enabling test–retest reliability estimation. The global severity score had excellent reliability (ICC = 0.98) while the nine component COAs all had good or excellent reliability (ICC > 0.75).

## 4 Discussion

Our suite of validated COAs (Saldaris et al. 2024; Saldaris et al. 2021; Ziniel et al. 2023; Saldaris et al. 2024; Saldaris et al. 2022; Saldaris et al. 2024; Saldaris et al. 2024) describes many facets of CDD, including functional abilities, seizures, vision, sleep, and other neurological and systemic features, representing the disease concept of interest for CDD and domains that are high priority for families (Mingorance et al. 2020; Neul et al. 2023). The SEM provided evidence that the COAs formed a coherent set of measures that captured the full diversity of global severity in CDD. Global severity scores correlated moderately well with an EEG biomarker, behavior, and quality of life providing further evidence for validity. It also had excellent test–retest reliability.

Our SEM had acceptable fit statistics and there were moderate correlations between global severity, EEG, and quality of life. There is a separate behavior domain of the CCSA‐Caregiver COA, but this was not included in our model as a potential component of severity. Greater motor and communication abilities which demonstrate less severity could enable difficult behaviors to be more readily expressed (Besag et al. 2016), and this was confirmed by a negative correlation between global severity and the behavior domain of the CCSA‐Caregiver. As such, we propose that problematic behavior should be a separate outcome to clinical severity when testing disease changing therapeutics. As part of this study, we had access to structured video data from many of the study subjects evaluating gross motor (Saldaris et al. 2024) and hand function (Saldaris et al. 2022). We recommend that videos should be collected as part of precision therapy clinical trials but not all trials will have the resources to include structured video data collection. Whilst these data would provide important information on clinical severity, we did not include scores from video assessments as part of the suite of observed variables in this SEM.

The phenotype of CDD is generally severe but there is variability in the frequency of seizures, motor, and communication abilities and other symptoms including insomnia and feeding difficulties (Leonard et al. 2022). As in any complex neurodevelopmental disorder, “severity” is a combination of the variety of symptoms that an individual exhibits, and the frequency and impact of each of those individual symptoms. Regardless of how symptoms combine, there is a sense from treating physicians, allied health professionals, and, most importantly, caregivers that a global concept of severity exists. Being able to quantify this global severity, while challenging, has merit for understanding relationships with the underlying biology and quality of life, and as a measure for disease modifying therapies. This approach has been suggested for similar rare neurodevelopmental disorders with heterogeneous and complex presentations (Tandon and Kakkis 2021). Domain measures provide insights into targeted effects of therapies and are needed for drugs that are hypothesized to target specific symptoms. However, we believe that a global severity score will have utility as an outcome measure in a clinical trial and this is supported by our results of applying a SEM to develop an algorithm, comprising a linear combination of component ratings, to generate a global severity score.

In preparation for clinical trials, we elected to evaluate COAs for functional abilities and associated neurological features, because of their impacts on the child and family (Mingorance et al. 2020; Neul et al. 2023). We administered and evaluated established COAs for sleep (Wetherby et al. 2002) and communication (Saldaris et al. 2024). We developed and modified the CDKL5 Severity Assessment (Demarest et al. 2019), using cognitive interviewing with clinicians and parent caregivers to form the CCSA‐Clinician (Saldaris et al. 2021) and the CCSA‐Caregiver respectively. This cognitive interviewing process began with the evaluation by clinicians and parent caregivers of features of existing measures as a starting point and subsequently diverged as these did not address their concerns. We then collected a large cross‐sectional dataset for psychometric evaluation to refine the item set, establish domains and evaluate reliability and validity. We followed the pipeline of COA development, modification, and evaluation drafted by the US Food and Drug Administration Guidance (Food and Drug Administration 2020; Food and Drug Administration 2022) and each COA had favorable psychometric properties. A combination of these measures should accurately capture the severity of disease in CDD and the rationale relates to the devastating effects of CDD on feeling and functioning.

The suite of COAs included parent caregiver reported measures. In the evaluation of individuals with intellectual disability, parent/proxy reports are relied upon both in research and clinical practice due to the limitations in affected individuals being able to report their own feelings and functioning. Collection of questionnaire data is an efficient and economical data collection method, which is another rationale for developing and using these measures. The clinician CCSA provided a supplementary perspective by contributing clinical observations on vision, communication, motor function, and muscle tone.

The global severity score correlated moderately well with a quantitative EEG feature contributing further evidence that EEG could be a useful biomarker of global severity (Saby et al. 2022; Saby et al. 2020). The moderate correlation between global severity score and QOL was expected but also confirms the relevance of the global severity score. We have previously identified that use of multiple anti‐seizure medications, functional impairments and poor sleep particularly influence QOL in CDD (Leonard et al. 2021). However, our global severity score did not include other known influencing factors such as pain. Pain is an important factor to caregivers (Reddihough et al. 2021) demonstrated in a sample of children with intellectual disability that also included some children with very severe impairments and multiple associated neurological conditions.

We acknowledge study limitations. Our analysis was based on correlations between observed variables to generate a global severity score and there were no comparisons to independent measures of severity for external validation. This is due to the very nature of the problem that COAs for individuals with severe DEEs such as CDD are not captured well by existing standardized assessments (Berg et al. 2024). As measures of convergent validation, the global severity score correlated moderately well with an EEG biomarker as well as quality of life. We did not have scores for all measures (such as CCSA‐Clinician for subjects evaluated remotely) for many individuals with CDD although we were able to take advantage of all available data, using Full Information Maximum Likelihood, by including individuals if they had two or more scores of any measure that contributed to any of the latent severity variables. In this way we were able to include ICDD data not accompanied by a clinical examination. We also acknowledge that some clinical features considered to be part of CDD, such as movement disorders that include chorea and stereotypies, were not included in our COAs and were therefore unable to influence the estimates of global severity. We found that these features, perhaps due to variabilities in clinical assessment, did not psychometrically correlate with each other or with other items in the CCSA‐Clinician (Saldaris et al. 2024); however, this may not be the case for other DEEs. Other DEEs can still benefit from our framework as a starting point for further validation in other specific disorders. We cannot explain whether the relationship between EEG and global severity score is linear or curvilinear perhaps because of the relatively small sample size of subjects with EEG data. Overall, with further studies, our approach may be generalizable across other DEE disorders which share symptoms and variation in phenotype with CDD, including very severe impacts (Scheffer et al. 2024). In support of this, we have extended the CCSA to STXBP1‐DEE (Abbott et al. 2025) and SYNGAP1‐DEE (Rong et al. 2023). We are currently collecting longitudinal data to understand COA stability, which will also provide opportunity to further evaluate the utility of the SEM models in calculating the global severity score.

## 5 Conclusion

SEM enabled analysis of the structural relationships between each of the measured and latent variables to generate a global severity score. Importantly, the model illustrates how the symptoms of CDD form a holistic and concretely measurable network of relationships that were otherwise intuitive. We propose that our set of COAs captures global severity in CDD and could indicate whether new precision therapies are successful in clinical trials as a primary or secondary outcome measure.

## Author Contributions

**Peter Jacoby**: conceptualization, writing–review and editing, methodology, data curation, formal analysis, validation, visualization. **Eric D. Marsh**: conceptualization, methodology, investigation, writing–review and editing, formal analysis, supervision, visualization. Scott **Demarest**: conceptualization, investigation, methodology, writing–review and editing, supervision, project administration. **Jacinta M. Saldaris**: conceptualization, writing–review and editing, investigation, formal analysis, project administration, data curation. Helen **Leonard**: conceptualization, investigation, methodology, writing–review and editing. **Heather E. Olson**: investigation, writing–review and editing, supervision. **Joni N. Saby**: investigation, methodology, validation, visualization, writing–review and editing, formal analysis, supervision, data curation. Elia **Pestana‐Knight**: investigation, writing–review and editing, supervision. Rajsekar **Rajaraman**: investigation, writing–review and editing, supervision. Dana **Price**: investigation, writing–review and editing, supervision. Judith **Weisenberg**: investigation, writing–review and editing, supervision. Bernhard **Suter**: investigation, writing–review and editing, supervision. Jenny **Downs**: conceptualization, investigation, funding acquisition, writing–original draft, methodology, validation, visualization, writing–review and editing, formal analysis, project administration, supervision. **Tim A. Benke**: conceptualization, investigation, funding acquisition, writing–review and editing, methodology, visualization, formal analysis, project administration, supervision.

## Conflicts of Interest

EM: The PI for Stoke therapeutics, Zogenix Pharmaceuticals, Acadia Pharmaceuticals, Marinus Pharmaceuticals, Takeda Pharmaceuticals, Epygenix Pharmaceuticals. He has received research support from NIH, Penn Orphan Disease center, International Rett syndrome foundation, International CDKL5 Research Foundation, Curaleaf Inc., is a consultant for Acadia Pharmaceuticals, and has prepared an educational program for Medscape.

SD: Consultancy for Biomarin, Neurogene, Marinus, Tysha, Ultragenyx, Zogenix, Capsida, Encoded, and Ovid Therapeutics. He has funding from the NIH, Project 8P and Mila's Miracle Foundation. He also serves on advisory boards for the non‐profit foundations Rare X, SLC6A1 Connect, Project 8P, Ring14 USA, FamilieSCN2A and *N* of 1 Collaborative.

HL: Consultancy for Marinus, Acadia, Avexis, Orion, Neurogene and Taysha; Clinical Trials with Anavex and Newron. All remuneration has been made to her department.

HO: Dr. Olson received consulting fees from Takeda Pharmaceuticals and Zogenix regarding clinical trial design, Ovid Therapeutics regarding clinical trial results, Marinus Pharmaceuticals regarding CDKL5 Deficiency Disorder, and has done consulting for the FOXG1 Research Foundation. She is an investigator for a trial with UCB Pharmaceuticals.

EP: Consultant for Acadia Pharmaceuticals for which she has received compensation.

RR: Speaker/consultant for Marinus Pharmaceuticals; consultant for UCB Pharmaceuticals; received funding from the International Foundation for CDKL5 Research.

DP: Consultancy for OVID therapeutics; clinical trials with Stoke, UCB, and Zogenix; all consultancies are unrelated to this work.

BS: Received research funding from the International Foundation for CDKL5 Research, Loulou Foundation, the National Institutes of Health, International Rett Syndrome Foundation, the Rett Syndrome Research Trust, and the Grace Science Foundation; consultancy for the IONIS pharmaceuticals, Neurogene, and Taysha; clinical trials with Acadia Pharmaceuticals Inc., Marinus Pharmaceuticals, Neurogene, and the Rett Syndrome Research Trust; all remuneration has been paid to Baylor College of Medicine.

JD: Consultancy for Marinus, Ultragenyx, Acadia, Avexis, Orion, Takeda, Neurogene and Taysha; clinical trials with Anavex and Newron; all consultancies are unrelated to this work and all remuneration has been made to her department.

TB: Received research funding from GRIN2B Foundation, the International Foundation for CDKL5 Research, Loulou Foundation, the National Institutes of Health, and Simons Foundation; consultancy for Alcyone, AveXis, GRIN Therapeutics, GW Pharmaceuticals, the International Rett Syndrome Foundation, Marinus Pharmaceuticals, Neurogene, Ovid Therapeutics, and Takeda Pharmaceutical Company Limited; clinical trials with Acadia Pharmaceuticals Inc., GW Pharmaceuticals, Marinus Pharmaceuticals, Ovid Therapeutics, and Rett Syndrome Research Trust; all remuneration has been made to his department.

PJ, JMS, JNS, and JW have no conflicts of interest.

## Funding

This study was supported by NIH‐NINDS U01NS114312 and the International Foundation for CDKL5 Research. TB is supported by the Children's Hospital of Colorado Foundation Ponzio Family Chair in Neurology Research. JD is supported by the Stan Perron Charitable Foundation. HEO is supported by 5K23NS107646‐05, 3K23NS107646‐05S1.

## Ethics Statement

Ethics approval for this study was provided by the Human Research Ethics Committees at the University of Colorado (COMIRB 19–2756) and University of Western Australia (RA/4/20/6198).

## Patient Consent Statement

Primary caregivers provided informed written consent to participate.

## Peer Review

The peer review history for this article is available at https://doi.org/10.1002/brb3.71061.

## Supporting information



Table S1. Summary of published reliability and validity for existing and novel outcome measures used for CDD.

## Data Availability

The data that support the findings of this study are not openly available but are available upon reasonable request from the corresponding author subject to IRB and governance approvals.
