# Supplementary material for: Beyond Seizures as an Outcome Measure: A Global Severity Scoring System for CDKL5 Deficiency Disorder
Source: Brain Behav. 2025 Nov 14;15(11):e71061. doi: 10.1002/brb3.71061 (PMC12616497; doi:10.1002/brb3.71061)
Supplement: Supplementary file 1 — Table S1. Summary of published reliability and validity for existing and novel outcome measures used for CDD. [file BRB3-15-e71061-s001.docx]

**Supplementary Table 1: Summary of published reliability and validity for existing and novel outcome measures used for CDD.**

|  |  | **Factor Analysis** | | | | | **Internal Consistency** | | **Average Variance Extracted** | **Divergent Validity** | **Test-retest Reliability** | **Inter-rater Reliability** | **Intra-rater Reliability** | **Known-groups validity** |
| --- | --- | --- | --- | --- | --- | --- | --- | --- | --- | --- | --- | --- | --- | --- |
| **Questionnaire** | **Domain** | N (used for analysis) | Factor Loadings | RMSEA | SRMR | Tucker-Lewis Index | Cronbach’s Alpha | Composite Reliability | AVE | Max Correlation Squared | ICC (N=18) | ICC (N=14)  (9 raters) | ICC (N=14)  (8 raters) | Significant Comparisons (p<0.05) |
| CCSA- Clinician [12] | Motor | 148 | All >= 0.89 | 0.116* | 0.092 | 0.968 | 0.85 | 0.96 | 0.84 | < AVE | 0.93 | 0.95 | All > 0.95 | Male v Female  Seizures v No seizures |
|  | Communication | 148 | All >= 0.81 |  |  |  | 0.89 | 0.93 | 0.76 | < AVE | 0.92 | 0.94 |  | Male v Female  Seizures v No seizures |
|  | Vision | 148 | All >= 0.57 |  |  |  | 0.80 | 0.88 | 0.59 | > AVE* | 0.88 | 0.85 |  | Male v Female |
| CCSA – Caregiver [12] | Seizures | 198 | All >= 0.52 | 0.078 | 0.096 | 0.954 | 0.80 | 0.89 | 0.54 | < AVE | 0.89 |  |  | Male v Female  Verbal v Non-verbal  Walks v Unable to walk |
|  | Alertness | 198 | All >= 0.63 |  |  |  | 0.83 | 0.90 | 0.59 | < AVE | 0.81 |  |  |  |
|  | Feeding | 198 | All >=0.88 |  |  |  | 0.90 | 0.95 | 0.87 | < AVE | 0.99 |  |  |  |
| SDSC [23] | Insomnia | 125 | All >= 0.52 | 0.152* |  | 0.821 | 0.78 | 0.82 | 0.48 | < AVE | 0.83 |  |  |  |
|  | Daytime Sleepiness | 125 | All >= 0.57 |  |  |  | 0.76 | 0.82 | 0.54 | < AVE | 0.87 |  |  | One or more AEDs v No AEDs |
| CSBS-DP ITC [8]  (1-factor) |  | 149 | All >=0.60 | 0.06 |  | 0.99 | 0.96 | 0.99 | 0.73 |  | 0.98 |  |  |  |

*sub-optimal values [28]

Abbreviations: CCSA - CDKL5 Clinical Severity Assessment

SDSC – Sleep Disturbance Scale for Children

CSBS-DP ITC - Communication and Social Behavior Scale

RMSEA – Root Mean Square Error of Approximation

SRMR – Standardized Root Mean Square Residual

ICC – Intra-class Correlation

AVE – Average Variance Extracted

AED – Anti-epileptic Drug
